# Intrinsic brain abnormalities in chronic rhinosinusitis associated with mood and cognitive function

**DOI:** 10.3389/fnins.2023.1131114

**Published:** 2023-03-10

**Authors:** Simin Lin, Miaomiao Nie, Bingshan Wang, Shaoyin Duan, Qianwen Huang, Naiming Wu, Zhishang Chen, Hengyu Zhao, Yi Han

**Affiliations:** ^1^Department of Radiology, Xiamen Cardiovascular Hospital of Xiamen University, School of Medicine, Xiamen University, Xiamen, China; ^2^Department of Radiology, Zhongshan Hospital of Xiamen University, School of Medicine, Xiamen University, Xiamen, China; ^3^Fujian Provincial Key Laboratory of Ophthalmology and Visual Science, Eye Institute of Xiamen University, School of Medicine, Xiamen University, Xiamen, China

**Keywords:** chronic rhinosinusitis, rs-fMRI, amplitude of low-frequency fluctuation, functional connectivity, depression, anxiety

## Abstract

**Background:**

Chronic rhinosinusitis (CRS) poses a risk for developing emotional and cognitive disorders. However, the neural evidence for this association is largely unclear. Resting-state functional magnetic resonance imaging (rs-fMRI) analysis can demonstrate abnormal brain activity and functional connectivity and contribute to explaining the potential pathophysiology of CRS-related mood and cognitive alterations.

**Methods:**

Chronic rhinosinusitis patients (CRS, *n* = 26) and gender- and age-matched healthy control subjects (HCs, *n* = 38) underwent resting-state functional MRI scanning. The amplitude of low-frequency fluctuations (ALFF) was calculated to observe the intrinsic brain activity. The brain region with altered ALFF was further selected as the seed for functional connectivity (FC) analysis. Correlation analysis was performed between the ALFF/FC and clinical parameters in CRS patients.

**Results:**

Compared with HCs, CRS patients exhibited significantly increased ALFF in the left orbital superior frontal cortex and reduced connectivity in the right precuneus using the orbital superior frontal cortex as the seed region. The magnitude of the orbital superior frontal cortex increased with inflammation severity. In addition, ALFF values in the orbital superior frontal cortex were positively correlated with the hospital anxiety and depression scale (HADS) scores. The ROC curves of altered brain regions indicated great accuracy in distinguishing between CRS patients and HCs.

**Conclusion:**

In this study, patients with CRS showed increased neural activity in the orbital superior frontal cortex, a critical region in emotional regulation, and this region also indicated hypoconnectivity to the precuneus with a central role in modulating cognition. This study provided preliminary insights into the potential neural mechanism related to mood and cognitive dysfunctions in CRS patients.

## Introduction

Chronic rhinosinusitis (CRS), one of the most common chronic diseases, is a set of disorders characterized by paranasal sinus and nose inflammation that lasts longer than 12 weeks (Gao et al., 2016). The prevalence of CRS is estimated to be more than 10% around the world based on the presence of symptoms or objective findings (Sedaghat et al., 2022) and reaches 13% of China’s population (Albu, 2020). CRS is a multi-factorial disease influenced by chronic inflammation, aberrant epithelial barrier and immunity, and imbalance of the nasal microflora (Sedaghat, 2017). There has been great advancement in the understanding of the pathophysiology of CRS: from the altered epithelial barrier and the epithelial-mesenchymal transition to the adaptive and innate immune system and, lastly, the effect of bacteria, including eosinophils and *Staphylococcus aureus*, on the persistence of disease (Bachert et al., 2020).

Among patients with CRS, the challenge is not only to constantly deal with CRS-related symptoms, such as nasal congestion and facial pain, but also a variety of potential complications, such as attention difficulties, possible depression and anxiety as recent reports (Ghadami, 2019; Kim et al., 2020; Jung et al., 2021). These symptoms may damage the patient’s quality of life, decrease the efficiency of work and incur serious medical costs (Gao et al., 2016). How CRS affects brain function is unclear. Previously proposed mechanisms mentioned that CRS-related brain function alterations is due to the effects of inflammatory cytokines on the brain (McAfoose and Baune, 2009; Jafari et al., 2021). Inflammatory cytokines may play a role in neurogenesis, neuromodulation and synaptic plasticity (McAfoose and Baune, 2009; Kim et al., 2016; Dehdar et al., 2019). The previous research demonstrated that inflammation might affect neural activity and neurogenesis (Fukushima et al., 2015; Salimi et al., 2021). The nasal epithelium is connected to the olfactory bulb via the olfactory nerve (Bohmwald et al., 2018). The mouse model studies of allergic rhinitis found neuroinflammation of the olfactory bulb (Lv et al., 2021). A recent study further discovered alterations in spontaneous brain activity in allergic rhinitis (Gao et al., 2021). We speculate that inflammatory cytokines in the upper respiratory tract, including CRS, enter the central nervous system via the olfactory nerve, which may lead to abnormal neural activity. Some studies also suggested that increased chronic inflammatory response in the nasal cavity may change the homeostasis of the local microbiome (Harrass et al., 2021). This microbial imbalance may affect the neuronal integrity of the central nervous system through mechanisms such as bacterial translocation to the central nervous system and/or modulation of the immune response in the central nervous system, leading to brain dysfunction (Hoggard et al., 2018; Harrass et al., 2021). Nevertheless, the pathophysiology of brain function alteration in CRS is still largely unknown.

Neuroimaging has proven to be a non-invasive tool for exploring the *in vivo* human brain, helping to detect the functional abnormalities in the brain at an early stage of various diseases (Lui et al., 2016). Resting-state functional magnetic resonance imaging (rs-fMRI) is a powerful and safe neuroimaging approach that inspects the changes in intrinsic neural activity via measurement of blood-oxygen level-dependent (BOLD) signal without task performance during the examination (Guo et al., 2022; Lin et al., 2022). Both amplitude of low-frequency fluctuation (ALFF) and regional homogeneity (ReHo) are crucial techniques to explore aberrant brain activity (Zang et al., 2004; Guo et al., 2022). The ALFF measures the low-frequency oscillation intensity of BOLD time courses in rs-fMRI (Zang et al., 2007; Guo et al., 2022). Although the exact biologic mechanisms of ALFF remain unclear, plenty of research has indicated that the ALFF changes are associated with local neuronal activity (Cui et al., 2017; Wang et al., 2019; Sun et al., 2022). ReHo measures the similarity in the time series of a given voxel to its nearest neighbors, which indicates that the ReHo alterations are associated with the coherence of spontaneous neuronal activity (Zang et al., 2004). Compared with ReHo, ALFF has the advantage of directly reflecting the intensity or amplitude of spontaneous brain activity (Qi et al., 2012). Resting-state functional connectivity (FC) measures interregional temporal correlation to reflect the intensity of interregional functional connectivity (Lu et al., 2017). Seed-based FC measures correlations of time series between selected seeds and the remaining voxels in the brain. This method is easy to measure and interpret. More to the point, it allows direct localization of the effect regions that show FC with the seed region at the whole-brain level (Gao et al., 2019; Liu et al., 2022). Some recent studies of chronic immune and inflammatory diseases, such as allergic rhinitis (Gao et al., 2021) and Crohn’s disease (Hou et al., 2019), used brain activity and functional connectivity to indicate abnormal brain regions associated with cognitive deficits or mood changes. Jafari et al. (2021) studied the functional connectivity of brain networks by independent component analysis and found altered functional connectivity in frontal medial cortices that regulated cognition function. However, to our knowledge, there are no reports on resting-state intrinsic neural activity in patients with CRS.

In this study, ALFF and seed-based FC methods are involved here for the first time to comprehensively explore the intraregional brain activity and interregional functional connectivity, attempting to provide potential information about the brain functional changes of CRS. We hypothesized that brain regions involved in emotional and cognitive functions might show abnormal brain activity and functional connectivity in patients with CRS, and altered brain regions may be associated with anxiety and depression.

## Materials and methods

### Participants

Twenty-six patients with CRS were recruited from the Zhongshan Hospital of Xiamen University from February to August 2022. The diagnostic criteria for CRS refer to the European Position Paper on Rhinosinusitis and Nasal Polyps (EP^3^OS) document (Fokkens et al., 2020). Inclusion criteria for patients of CRS included (1) the Lund-Mackay scoring (LMS) ≥ 8; (2) the age range was between 20 and 50. Thirty-eight gender- and age-matched healthy volunteers were also recruited from the local community as a healthy control group. The exclusion criteria for all subjects were (1) serious anxiety or depression (HADS-A or HADS-D scores more than 14; (2) history of brain surgery, tumor, or neuropsychiatric disease; (3) history of alcohol or drug abuse; (4) left-handed; (5) contraindications to MR examinations. The experiment was authorized by the Medical Ethics Committee of Zhongshan Hospital of Xiamen University. Written informed consent was obtained from participants enrolled in this study.

Demographic and clinical parameters such as sex, age, education level, disease course, clinical symptoms, medical and surgical history, and complications were collected. CRS severity was also calculated using objective and subjective scores, including the Lund-Mackay score (LMS) and the visual analog scale (VAS) score. In addition, we collected the hospital anxiety and depression scale (HADS) scores of each subject.

### MRI parameters

Images were obtained using a Philips Ingenia 3.0 T CX (Philips Healthcare, Best, Netherlands) at the Zhongshan Hospital of Xiamen University. Each subject was instructed to close their eyes, keep awake, try not to think and remain breathing calmly during the entire scanning process. Earplugs and foam pads were applied to minimize head motion and noise during scanning.

Resting-state functional MR imaging, including 200 volumes, was acquired by a gradient-echo-planar imaging sequence (EPI) in the axial plane with the following parameters. echo time (TE) = 25 ms; repetition time (TR) = 2,000 ms; flip angle (FA) = 65°; thickness = 2.5 mm; field of view (FOV) = 219 mm × 219 mm; matrix = 88 × 88; gap = 0 mm; voxel size = 2.5 mm × 2.5 mm × 2.5 mm; number of slices = 57.

Structural images including high-resolution T1-weighted and T2-weighted brain images. T1-weighted scans were obtained using a 3D magnetization-prepared rapid gradient-echo (MP-RAGE). The parameters are as follows: TE = 3.0 ms; TR = 6.6 ms; FA = 8°; thickness = 1.0 mm; FOV = 240 mm × 240 mm; matrix = 240 × 240; voxel size = 1.0 mm × 1.0 mm × 1.0 mm; gap = 0 mm; number of slices = 180. T2-weighted structural imaging was used to calculate all sinonasal Lund-Mackay scores of each patient by two experienced radiologists. Prior studies have already shown a good correlation between computed tomography and magnetic resonance imaging-based scores (*R* = 0.837; *P* < 0.001) (Lin and Bhattacharyya, 2009; Bhattacharyya, 2010). The parameters of the SPIR T2-weighted images: TE = 280 ms; TR = 3,000 ms; FA = 90°; thickness = 1.0 mm; matrix = 240 × 240; number of slices = 320.

### Data preprocessing

Resting-state BOLD data were preprocessed using Data Processing and Analysis for Brain Imaging (DPABI). This toolbox is based on Statistical Parametric Mapping 12 (SPM12). The preprocessing included the following steps: the first 10 volumes for each participant were abandoned to minimize the instability of initial imaging signals. The remaining volumes were corrected for slice timing and then realigned to correct head motions. Each subject who exhibited a rotational or translational motion parameter more than 2° or 2 mm was discarded. Each functional volume was spatially normalized to the Montreal Neurological Institute template using the DARTEL technique and resampled to 3-mm voxels. Subsequently, the images were smoothed using a 6 mm full-width-at half maximum (FWHM) Gaussian kernel. After smoothing, nuisance variables were regressed out from the data, including linear drift, cerebrospinal fluid signal, white matter signal, and Friston 24-parameter head motion parameters. Finally, the band-pass filtering (0.01–0.08 Hz) was performed to remove the influence of physiological noise.

### ALFF and seed-based FC analyses

Amplitude of low-frequency fluctuations and FC analyses were calculated via REST software. For ALFF analysis, after preprocessing, the fast Fourier transform was applied to transform the time series to the frequency domain (FFT). Then the square root of the power spectrum was computed, and the averaged square root was obtained across 0.01-0.08 Hz to acquire the ALFF. Eventually, to minimize the global influence, the ALFF values were divided by the global mean ALFF values for standardization. Based on the findings of ALFF, the abnormal brain region was selected as the seed. A radius of 5 mm around the peak MNI was used as the region of interest (ROI) for the seed-based FC analysis. Pearson correlation coefficients were conducted between the time courses of seed regions and the time series of all voxels in the entire brain. Fisher’s z-transform was performed on the Pearson correlation coefficients to generate an approximately normal distribution for further statistical analysis.

### Statistical analysis

Demographics and clinical parameters between the two groups were calculated using SPSS 26.0. An independent two-sample t-test was performed for continuous variables, and a chi-squared test was performed for proportions.

Independent two-sample t-tests were performed for differences in ALFF and FC of each voxel between the CRS and HCs using SPM12 software. Sex, age, and levels of education were imported as covariates to minimize the impacts of confounding covariates. The cluster-level False Discovery Rate (FDR) method was used for multiple comparison correction, with a cluster-defined threshold of *P* = 0.001 and a corrected cluster significance of *P* < 0.05. The HADS-A and VAS scores do not conform to the normal distribution, so Spearman correlation analysis was used to analyze the relationship between ALFF/FC and HADS-A and VAS. Pearson correlation analysis was performed to investigate the relationship between ALFF/FC and HADS-D scores and LMS.

To evaluate the diagnostic potential of ALFF and FC values in abnormal brain regions in separating CRS patients from HCs, we also used the receiver operating characteristic (ROC) curve for analysis. Besides, the level of precision was quantified by calculating the area under the curve.

## Results

### Demographics and clinical characteristics

A total of 26 patients with CRS and 38 HCs aged 20–50 years were included in the analysis, with no significant differences in gender (χ^2^ = 1.546, *P* = 0.214), age (*t* = −1.250, *P* = 0.216), HADS total scores (*t* = −0.966, *P* = 0.338), HADS-A (*t* = −1.112, *P* = 0.270) and HADS-D (*t* = −0.489, *P* = 0.626) scores between the two groups. There is a statistical difference between the two in the level of education (*t* = 3.231, *P* = 0.002). The disease duration of the patients varied widely, from a minimum of 6 months to a maximum of more than 20 years, resulting in a relatively large standard deviation. Details are summarized in Table 1.

**TABLE 1:** Demographic and clinical parameters.

	CRS patients	HCs	Statistics	*P*-value
	(*n* = 26)	(*n* = 38)		
**Demographic characteristics**				
Gender: Men (%)	19 (73.1%)	22 (57.9%)	χ^2^ = 1.546	0.214^b^
Age (year)	36.88 ± 7.63	34.45 ± 7.69	*t* = −1.250	0.216^a^
Education (year)	14.00 ± 3.15	16.16 ± 2.20	*t* = 3.231	0.002^a^
**Clinical characteristics**				
Disease duration (year)	8.08 ± 7.63	N/A	N/A	N/A
Nasal obstruction/congestion	24 (92.3%)	N/A	N/A	N/A
Nasal discharge	19 (73.1%)	N/A	N/A	N/A
Hyposmia/anosmia	8 (30.8%)	N/A	N/A	N/A
Facial pain/pressure	7 (26.9%)	N/A	N/A	N/A
Asthma	2 (7.7%)	N/A	N/A	N/A
Nasal polyp	11 (42.3%)	N/A	N/A	N/A
Previous sinonasal surgery	7 (21.9%)	N/A	N/A	N/A
LMS	11.38 ± 2.53	N/A	N/A	N/A
**Questionnaires**				
HADS total scores	9.46 ± 5.85	8.16 ± 4.90	*t* = −0.966	0.338^a^
HADS-A scores	5.12 ± 3.50	4.26 ± 2.63	*t* = −1.112	0.270^a^
HADS-D scores	4.35 ± 3.12	3.97 ± 2.90	*t* = −0.489	0.626^a^
VAS	5.63 ± 1.81	N/A	N/A	N/A
**Medicine in three months**				
Nasal glucocorticoid	15 (57.7%)	N/A	N/A	N/A
Antibiotics	4 (15.4%)	N/A	N/A	N/A

The data are shown as the mean values ± standard deviations.

^a^The *P*-value was obtained by two-sample *t*-test.

^b^The *P*-value was obtained by Chi-square test.

N/A, not applicable; LMS, Lund-Mackay scoring; HADS, hospital anxiety and depression scale; HADS-A, hospital anxiety and depression scale-anxiety; HADS-D, hospital anxiety and depression scale-depression; VAS, visual analogue scale.

### ALFF and seed-based FC analyses

**FIGURE 1:**
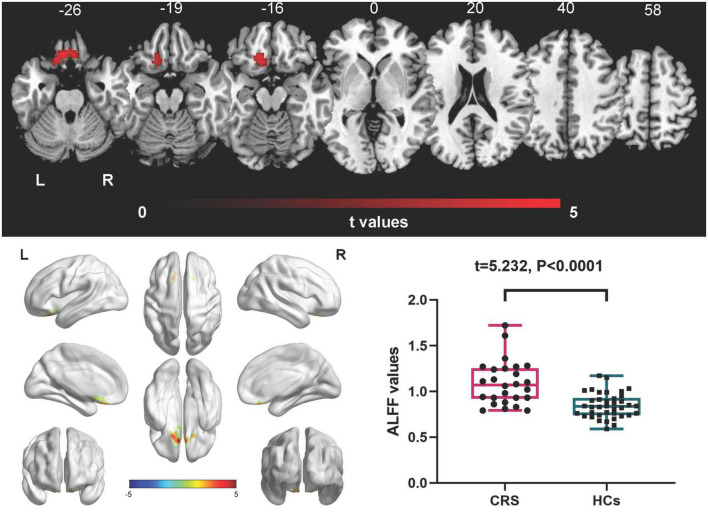
Amplitude of low-frequency fluctuations (ALFF) differences between CRS patients and HCs (*P* < 0.05, FDR corrected). Compared with HCs, patients with CRS showed significantly increased ALFF in the left orbital superior frontal cortex, extending to the left rectus. L, left; R, right.

**FIGURE 2:**
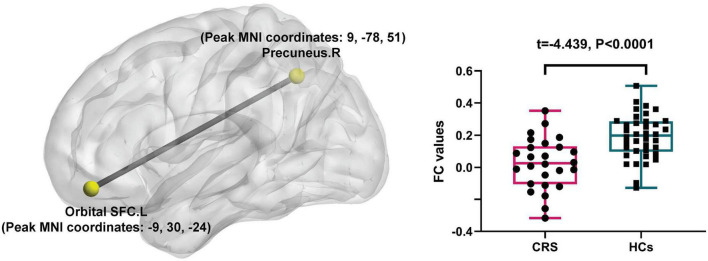
Seed-based FC differences between CRS patients and HCs (*P* < 0.05, FDR corrected). Compared with HCs, patients with CRS showed significantly decreased FC between the seed region in the left orbital superior frontal cortex and the right precuneus. SFC, superior frontal cortex; L, left; R, right.

In comparison with the HCs, patients with CRS showed significantly increased ALFF values in the left orbital superior frontal cortex extending to the left rectus (cluster size of 130 voxels, *t* = 5.0765, *P* < 0.05, FDR corrected) (Figure 1). FC in the right precuneus was found to be decreased when the left orbital superior frontal cortex was used as the seed point (with a cluster size of 68 voxels, *t* = −4.0840, *P* < 0.05, FDR corrected) (Figure 2). Details are shown in Table 2.

**TABLE 2:** Differences in ALFF and FC values between CRS patients and HCs.

Brain regions	Peak MNI coordinates			BA	Cluster size	*t*-Value
	x	y	z			
**ALFF differences**						
Left orbital SFC, extending to left rectus	−9	30	−24	11	130	5.0765
**FC differences**						
Right precuneus	9	−78	51	7	68	−4.084

x, y, and z are the locations of the peak voxels in standard MNI coordinates. FDR correction, cluster-level: *P* < 0.05. SFC, superior frontal cortex; MNI, Montreal Neurological Institute; BA, Brodmann area.

### Correlation analysis

**FIGURE 3:**
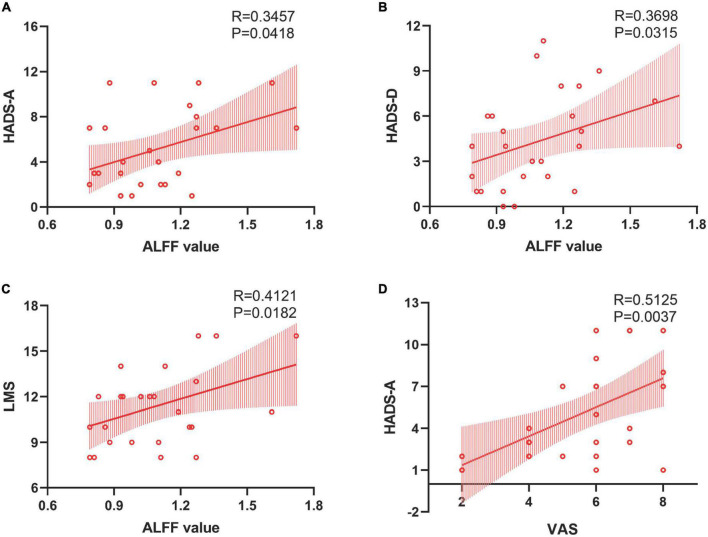
Correlations between the ALFF value in the left orbital SFC and clinical assessment. Panels **(A,B)** revealed positive correlations between ALFF in the left orbital SFC and HADS-A and HADS-D scores. Panel **(C)** established a “dose-dependent” association between the ALFF value in the left orbital SFC and the objective severity of inflammation. Panel **(D)** showed a positive correlation between the patient’s subjective severity of inflammation and anxiety scores. SFC, superior frontal cortex; HADS-A, hospital anxiety and depression scale-anxiety; HADS-D, hospital anxiety and depression scale-depression; LMS, Lund-Mackay scoring.

In patients with CRS, ALFF in the orbital superior frontal cortex was found to be positively correlated with the HADS-A scores (*R* = 0.3457, *P* = 0.0418, Figure 3A), HADS-D scores (*R* = 0.3698, *P* = 0.0315, Figure 3B) and LMS (*R* = 0.4121, *P* = 0.0182, Figure 3C), as well as a significant correlation was found between VAS and HADS-A scores (*R* = 0.5125, *P* = 0.0037, Figure 3D). However, there was no significant correlation between FC alterations in the right precuneus and clinical index, including HADS scores, LMS and VAS. Moreover, the correlation analysis between disease duration and both ALFF and FC values showed no statistical difference.

### Receiver operating characteristic curve

**FIGURE 4:**
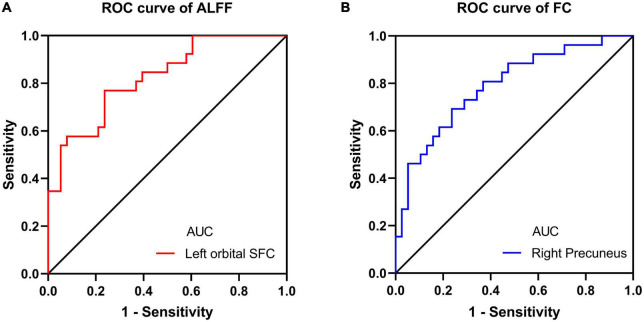
ROC curve analysis for altered brain region. **(A)** The AUC of the ALFF values of the left orbital SFC was 0.8229 (*P* < 0.001; 95% CI: 0.7211–0.9247); **(B)** the AUC of the FC values of the right precuneus was 0.7895 (*P* < 0.001; 95% CI: 0.6762–0.9028). ROC, receiver operating characteristic; AUC, area under the curve; CI, confidence interval; SFC, superior frontal cortex.

We also performed the ROC curve analysis of mean ALFF and FC values in these changed brain regions to find potential imaging biomarkers to separate CRS patients from healthy controls. The area under the ROC curve (AUC) of the ALFF values of the left orbital superior frontal cortex and the FC values of the right precuneus were 0.8229 (Figure 4A) and 0.7895 (Figure 4B), respectively. It showed good accuracy in distinguishing between CRS patients and HCs.

## Discussion

This study presents preliminary neurological evidence for ALFF and FC abnormalities as a potential basis for emotional and cognitive alterations in patients with CRS. We found that compared with healthy controls, patients with CRS presented enhanced ALFF values in the left orbital superior frontal cortex extending to the left rectus, both belonging to the orbitofrontal cortex (OFC), which is involved in emotion regulation and decision-making (Schoenbaum et al., 2006; Subramaniam et al., 2016). This brain region demonstrated decreased functional connectivity to the precuneus, which is prominent for its unique role in cognitive modulation (Cavanna and Trimble, 2006). The severity of inflammation as well as anxiety and depression problems are significantly positively correlated with spontaneous neural activity in the OFC. Taken together, these findings provide a neuropathophysiological basis for understanding the relationship between CRS and the increased risk of emotional and cognition dysfunctions, which contribute to clinical prevention and treatment for improving the life quality of patients.

The orbitofrontal cortex, as a part of the prefrontal cortex, is implicated in emotion regulation and decision-making (Schoenbaum et al., 2006; Subramaniam et al., 2016; Burks et al., 2018). The OFC receives environmental irritation, affective information, and emotional or social responses (Cheng et al., 2016). Banerjee et al. (2020) indicated that the orbitofrontal cortex of the cerebral cortex reprograms neurons located in sensory areas, allowing humans to make flexible decisions. Wikenheiser et al. (2017) found that OFC neurons encode the expected outcome of a decision task by recording neuronal activity in the OFC during the execution of the decision task in mice. A theory of depression has been developed that depression may be related to the over-responsiveness of the OFC to the non-reward or punishment system (Milad and Rauch, 2007). Neuroimaging has shown evidence of altered brain activity or functional connectivity in the OFC of patients with depression and anxiety disorders. For instance, rs-fMRI in healthy male participants revealed that trait anxiety is associated with fALFF in the medial OFC and the functional connectivity between the medial OFC and precuneus (Xue et al., 2018). Depression patients showed increased regional cerebral blood flow, higher ALFF, lower functional connectivity, reduced cortical thickness and gray matter volume in the OFC (Lyoo et al., 2004; Price and Drevets, 2010; Xue et al., 2018; Gong et al., 2020). A cohort study indicated the overall incidence of depression and anxiety disorders was increased in CRS patients than in healthy people during the 11-year follow-up (Kim et al., 2019). We speculate that increased brain activity in the OFC involved in emotion regulation in patients with CRS may be associated with anxiety and depression. Although HADS scores were higher in patients with CRS, we did not find a statistical difference between groups in scores of anxiety and depression. Thus, given the brain’s ability to adapt and compensate, especially among young individuals excluding severe anxiety and depression, our results may present early and subclinical intrinsic brain abnormalities (including ALFF and FC) that may precede or be more sensitive than clinical symptoms. To further support our speculation, we collected several clinical indexes for CRS patients to investigate the association between CRS and mood disorders. As expected, The ALFF values of OFC increase with the severity of inflammation, which indicates a dose-dependent association between neuronal activity in OFC and the degree of inflammation. This study also showed a significantly positive correlation between neuronal activity in OFC of CRS patients and scores of anxiety and depression, and we guess that sinusitis patients have overreaction of OFC to non-rewards, which may increase the incidence of mood disorders. Relating brain activity abnormalities in OFC to HADS scores may help to provide new insights into the relationship between CRS and anxiety and depression.

The precuneus, as a functional core of the default mode network, plays an important role in cognitive processing (Li et al., 2019). The precuneus is implicated in the representation of self-related processing, autobiographical memory, and visuospatial processing (Cavanna and Trimble, 2006; Zhang and Li, 2012). Besides, the neuroimaging studies had consistently identified that the precuneus was activated when subjects were triggered with a higher action identification level, which is powerful evidence of the connection of the precuneus to higher-order cognitive functions such as abstract mental imagination and attention shifting (Lou et al., 2004; Simon et al., 2004). Jafari et al. (2021) used independent component analysis to indicate altered brain connectivity within the frontoparietal and default mode networks, both of which play a critical role in cognition modulation. In contrast to us, their findings are based on a cohort of young participants identified from public databases, lacking relevant clinical history and not suitable to represent a clinical CRS population. A large sample functional connectivity study found that the precuneus is strongly associated with the effects of depressive problems (Cheng et al., 2018). It is supposed that the precuneus is involved in the sense of self, and depression is associated with an impaired representation of self. These findings together support our points that hypoconnectivity between OFC and precuneus may contribute to explaining the increased risk of mood and cognitive impairments of patients with CRS.

Given the evidence of chronic rhinosinusitis associated with mood and cognitive function, we make the following assumptions about the pathogenesis: (1) the physiological effect of nasal congestion and its influence on sleep quality may subsequently affect psychiatric symptoms negatively, such as anxiety and depression (Fang et al., 2010); (2) pro-inflammatory cytokines can enter the central nervous system and interact with a cytokine network in the brain to affect brain function (Capuron and Miller, 2011); (3) an imbalance of local microbiome homeostasis may affect the neuronal integrity of the central nervous system and cause brain-related symptoms (Harrass et al., 2021). Further study should focus on the specific regulatory mechanisms of molecules between the sinonasal inflammation and abnormal brain function aiming to find a way to block the vicious circle.

## Limitation

Several limitations are mentioned in this study. The sample size of this research was relatively small, which may reduce statistical power and limit the further investigation of the relationship between the abnormal activity in the brain regions and CRS. Furthermore, we deduced that CRS was associated with cognitive dysfunction. However, the cognitive analysis using professional scales or questionnaires did not conduct here, which may influence the credibility of the results. And the brain functional abnormal changes in CRS is a crucial project that deserves much further exploration.

## Conclusion

This study demonstrated the intrinsic abnormal brain activity in the orbital superior frontal cortex in chronic rhinosinusitis patients, which is associated with mood disorders, including depression and anxiety. Besides, the functional connectivity alterations between OFC and the precuneus in CRS patients. This rs-fMRI study provides preliminary evidence for alterations in brain activity and functional connectivity as a potential basis for mood and cognitive dysfunction in patients with CRS.

## Data availability statement

The raw data supporting the conclusions of this article will be made available by the authors, without undue reservation.

## Ethics statement

Written informed consent was obtained from the individual(s) for the publication of any potentially identifiable images or data included in this article.

## Author contributions

SL searched the literature, analyzed the data, and wrote the first version of the manuscript. MN and SD collected the clinical information, questionnaire scales, and imaging data of patients. BW and QH collected the clinical information, questionnaire scales, and imaging data of healthy controls. NW and ZC created tables and figures. YH and HZ revised the manuscript for critical intellectual content. All authors read and approved the final manuscript.
